# Contractile dysfunction and nitrergic dysregulation in small intestine of a primate model of Parkinson’s disease

**DOI:** 10.1038/s41531-019-0081-9

**Published:** 2019-06-10

**Authors:** Erika Coletto, John S. Dolan, Sara Pritchard, Alex Gant, Atsuko Hikima, Michael J. Jackson, Christopher D. Benham, K. Ray Chaudhuri, Sarah Rose, Peter Jenner, Mahmoud M. Iravani

**Affiliations:** 1 Neurodegenerative Disease Research Group, Institute of Pharmaceutical Sciences, Faculty of Life Sciences and Medicine, King’s College London, London, UK; 2 Department of Clinical and Pharmaceutical Sciences, University of Hertfordshire, Hatfield, UK; 3 Basic and Clinical Neuroscience, The Maurice Wohl Clinical Neuroscience Institute, Kings College London, London, UK

**Keywords:** Neurotransmitters, Constipation

## Abstract

Bowel dysfunction is a common non-motor symptom in Parkinson’s disease (PD). The main contractile neurotransmitter in the GI tract is acetylcholine (ACh), while nitric oxide (NO) causes the relaxation of smooth muscle in addition to modulating ACh release. The aim of this study was to characterise functional and neurochemical changes in the isolated ileum of the 1-methyl-4-phenyl-1,2,3,6-tetrahydropyridine (MPTP)-treated marmoset, an established model of PD motor dysfunction. While NO-synthase inhibitor L-NAME concentration dependently augmented the neurogenically-evoked contractions and inhibited the relaxations in normal tissues, it had no effects on the MPTP ileum. Immunohistochemical analyses of the myenteric plexus showed that ChAT-immunoreactivity (-ir) was significantly reduced and the density of the enteric glial cells as shown by SOX-10-ir was increased. However, no change in TH-, 5-HT-, VIP- or nNOS-ir was observed in the MPTP tissues. The enhancement of the neurogenically-evoked contractions and the inhibition of the relaxation phase by L-NAME in the control tissues is in line with NO’s direct relaxing effect on smooth muscle and its indirect inhibitory effect on ACh release. The absence of the relaxation and the inefficacy of L-NAME in the MPTP tissues suggests that central dopaminergic loss dopamine may eventually lead to the impairment of NO signal coupling that affects bowel function, and this may be the result of a complex dysregulation at the level of the neuroeffector junction.

## Introduction

Parkinson’s disease (PD) encompasses a variety of motor and non-motor symptoms. Autonomic dysfunction particularly of the gastrointestinal tract (GI), is a common non-motor problem in PD that may precede motor symptoms by more than 10 years before individuals are diagnosed.^1^ Difficulties in swallowing, constipation, dysphagia, and delayed gastric emptying and reduced gastric motility are core components of this dysautonomia which may get worse as the disease progresses.

GI dysfunction in PD may have a central origin subsequent to the degeneration of nigro-striatal dopaminergic neurones or an intrinsic origin at the level of the enteric nervous system. A range of enteric neurotransmitters/neuromodulators including acetylcholine (ACh), serotonin (5-HT), dopamine (DA), noradrenaline (NA), vasoactive intestinal peptide (VIP) and nitric oxide (NO) control smooth muscle activity. However, it is not clear whether single or multiple neurotransmitters are involved in the control of the smooth muscle contractility and gut motility or how these change in relation to PD. Rodent studies using unilateral 6-OHDA lesioning of the dopaminergic nigro-striatal tract have overall shown no significant change in the level of ChAT-immunoreactivity (-ir) throughout the GI tract following 6-OHDA in the rat^2–4^ or in the MPTP-treated primate model of PD.^5^ An increase in VIP-ir and a decrease in nNOS-ir was detected^3,4^ in the 6-OHDA rat model while in the MPTP treated mouse no change in either NADPH-diaphorase staining or ChAT-ir was observed compared to the controls—although the level of TH-ir was reduced in the myenteric plexus of the ileum.^6^ In contrast to the rodent studies, in the MPTP treated primate,^5^ an increase in nNOS-ir in the myenteric plexus and the submucosal plexus of the colon was observed. So, species-related differences and their differing sensitivity to MPTP or to a more targeted central 6-OHDA-induced dopaminergic lesion may contribute to the pattern of change observed.

In contrast, very little is known about small-intestine motility in PD. To date only a few studies have investigated colonic motility in experimental models of the disease^2–4,6–10^ and no study has so far investigated the functional consequences of nigro-striatal dopaminergic neuronal loss in the small intestine of either rodents or primates. Colon is the main site of water and solute absorption which in addition to dysmotility contribute to constipation, but few studies have looked at the ileum where poor gastric transit is thought to be a contributory factor to constipation.^11,12^ The present study investigates the spontaneous contractile and neurogenically driven contractile and the relaxant responses of the isolated common ileum in normal and MPTP-treated common marmoset. Alterations in response have been correlated to changes in those neurotransmitters and neuromodulators involved in contractile and relaxant responses. An important aim was to study the impact of NOS inhibition on the contractile response of the normal and MPTP-treated marmoset ileum following neurogenic stimulation using electrical field stimulation as NO is considered to be a key player in the control of both the contractile and relaxant.^13–15^

## Results

### MPTP-induced loss of TH-ir in the nigrostriatal tract

**Fig. 1:**
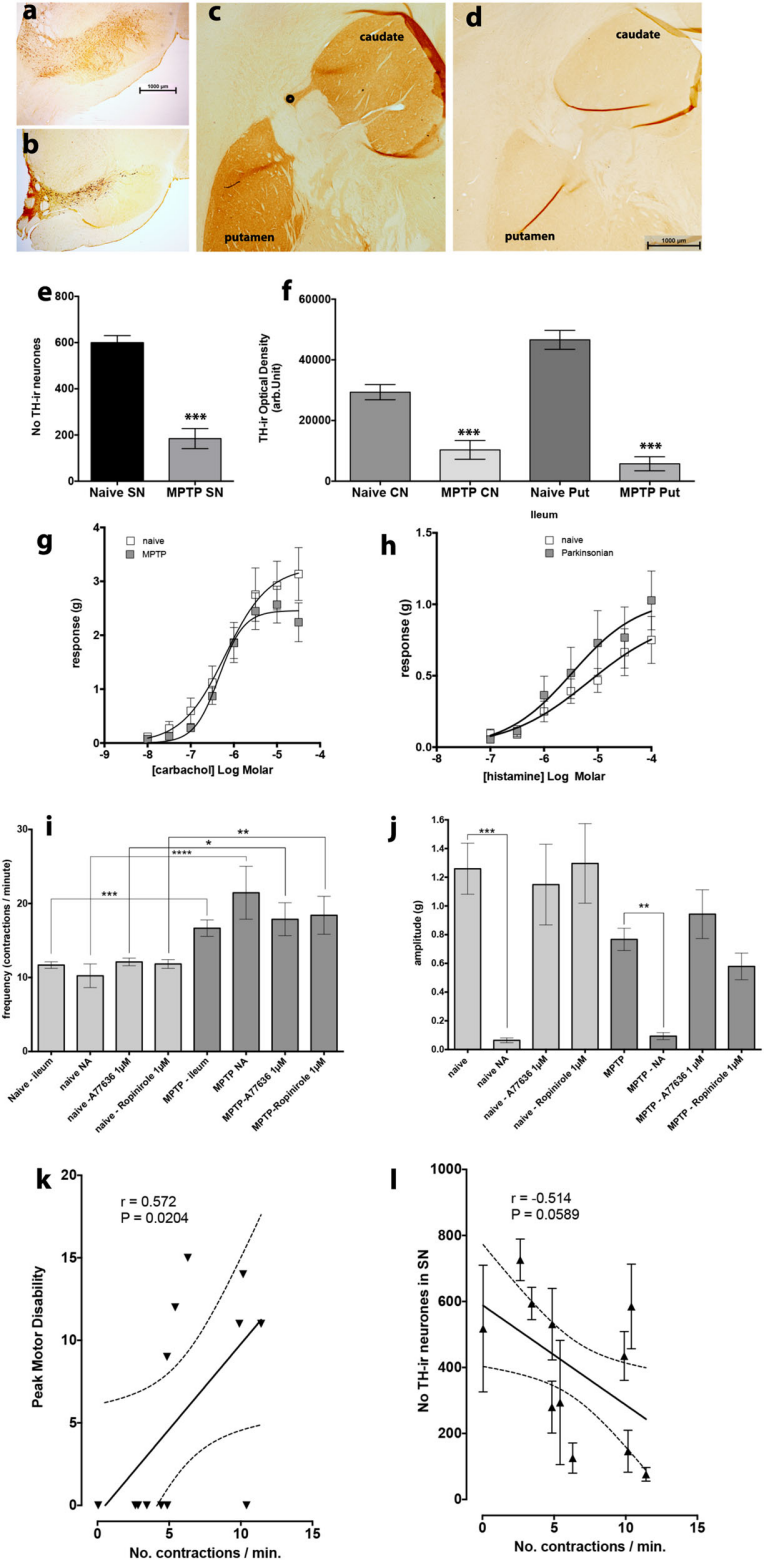
The relationship between nigrostriatal tract TH-ir and contractile responses of the ileum. The effect of MPTP treatment on TH-ir in the substantia nigra (SN, **a**, **b, e**) and the caudate nucleus and putamen **c**, **d, f** and the effect of lesioning on agonist evoked and spontaneous contractile response of ileum. MPTP treatment resulted in marked reduction of TH-ir neurones **e** and TH-ir nerve terminals in the caudate nucleus and the putamen **f**. Agonist evoked and spontaneous contractile responses of the normal, drug naive ileum (open data points) and the ileum obtained from MPTP (shaded data points) treated common marmosets. Administration of carbachol **g** and histamine **h** evoked a concentration dependent contraction of the isolated ileum which were not significantly different between the normal ileum and those from MPTP treated animals. However, the frequency of spontaneous activity was significantly larger in preparations obtained from MPTP animals **i** while the amplitude of spontaneous contractions appeared lower **j** although this reduction did not reach statistical significance. Administration of noradrenaline (NA) or D1 (A77636) or D2 (ropinirole) agonists affected the frequency and the amplitude of the spontaneous contractions similarly. Panels **k** and **l** show correlation between the frequency of contraction (number of contraction/min) and motor disability **k** and the number of TH-ir neurones in the SN. Each bar is the mean ± s.e.m. of *n* = 7. *P < 0.05; **P < 0.001; ***P < 0.0005

There was marked loss of tyrosine hydroxylase immunoreactivity (TH-ir) in both the substantia nigra (SN) and in the nerve terminal regions in the MPTP-treated animals (Fig. 1a–f). Following MPTP treatment the TH-ir neurones in the SN were significantly reduced by approximately 70% (*P* < 0.0001, unpaired *t*-test *n* = 5–6). The TH-ir in the caudate nucleus and the putamen were similarly affected (*P* < 0.0001; one-way ANOVA, *F* = 3,22; caudate, *n* = 7; MPTP putamen, *n* = 3–7).

### Effect of agonists on contractile response

There was no significant difference in the concentration dependent contraction to carbachol of both normal and MPTP ileum preparations (Fig. 1g). The carbachol concentration response curve produced LogEC_50_ of −6.641 to −5.755 and −6.558 to −6.128 for the normal and MPTP ileum, respectively. Similarly, cumulative application of histamine (100 nM to 100 µM) produced concentration dependent contraction of both normal and MPTP ileum but compared to carbachol the contractions were markedly smaller in magnitude. No difference in histamine concentration response curves was seen between the normal and the MPTP tissues with LogEC_50_ of −6.68 to −3.636 and −6.58 to −4.371 for the normal and MPTP ileum, respectively (Fig. 1h). The slopes of the carbachol and histamine concentration/response were however different regardless of the animal treatment.

### Noradrenaline and dopamine agonists effects on spontaneous activity

Administration of noradrenaline (NA, 10 µM) produced relaxation of the basal resting tone of the smooth muscle in both normal and MPTP ileum. In the normal ileum samples, NA reduced the baseline by −0.5 ± 0.09 while in the MPTP ileum baseline reduction was −0.3 ± 0.07 g, but this difference was not statistically significant (*P* = 0.116; *n* = 7).

The frequency of spontaneous contractions of ileum was significantly increased by 30–40% following MPTP treatment (Fig. 1i) but the amplitude was non-significantly reduced (Fig. 1d). Administration of NA, dopamine D1 agonist, A77636 (1 µM) or D2 Agonist, ropinirole (1 µM) did affect the frequency of spontaneous contractions of normal or MPTP ileum. However, only NA significantly reduced the amplitude of the spontaneous contractions in both normal and MPTP ileum (Fig. 1j). There were no differences between the effect of NA on the normal or MPTP tissues.

Overall there was a positive correlation between motor disability (see Table 1) and a negative correlation between the number of TH-ir and the frequency of spontaneous activity. However, in the latter this relationship failed to reach statistical significance (*P* = 0.0589; Fig. 1k, l).

**Table 1:** The details of common marmosets used in the study

Animal ID	Sex	Treatment	Survival (days) following MPTP	Peak motor disability score
**KCL14**	Female	N	n/a	0
**H26**	Female	N	n/a	0
**Y007**	Male	N	n/a	0
**X046**	Male	N	n/a	0
**Y107**	Female	N	n/a	0
**9094**	Male	N	n/a	0
**9102**	Female	N	n/a	0
**KCL10**	Female	MPTP	202	12.0
**KCL4**	Male	MPTP	169	11.0
**Px48**	Female	MPTP	1123	9.0
**8036**	Female	MPTP	494	11.0
**X036**	Male	MPTP	169	14.0
**XO53**	Male	MPTP	521	15.0
**XO78**	Male	MPTP	358	14.0

This table shows animal identification tags, their sex, their treatment and survival time following MPTP treatment (0.2 mg/kg for 5 consecutive days) and their peak stable motor disability. Basal motor disability was assessed during the 1 h acclimatisation period using an established motor disability rating scale: alertness (normal 0, sleepy 2); reaction (normal 0, reduced 1, slow 2, absent 3); checking movements (present 0, reduced 1, absent 2); attention and eye movements (normal 0, abnormal 1); posture (normal 0, abnormal trunk +1, abnormal limbs +1, abnormal tail +1, or grossly abnormal 4); balance/co-ordination (normal 0, impaired 1, unstable 2, spontaneous falls 3); vocalisation (normal 0, reduced 1, absent 2); motility (normal 0, bradykinesia/hyperkinesia 1, akinesia/hyperkinesia 2).^30^ A maximum score of 18 indicated a high degree of motor disability and a score of 0 indicated normal behaviour. The end of the survival time, is when animals were terminally anaesthetised and the ilea and brains were dissected

### Effect of NOS-inhibitors on EFS-evoked contractile responses

**Fig. 2:**
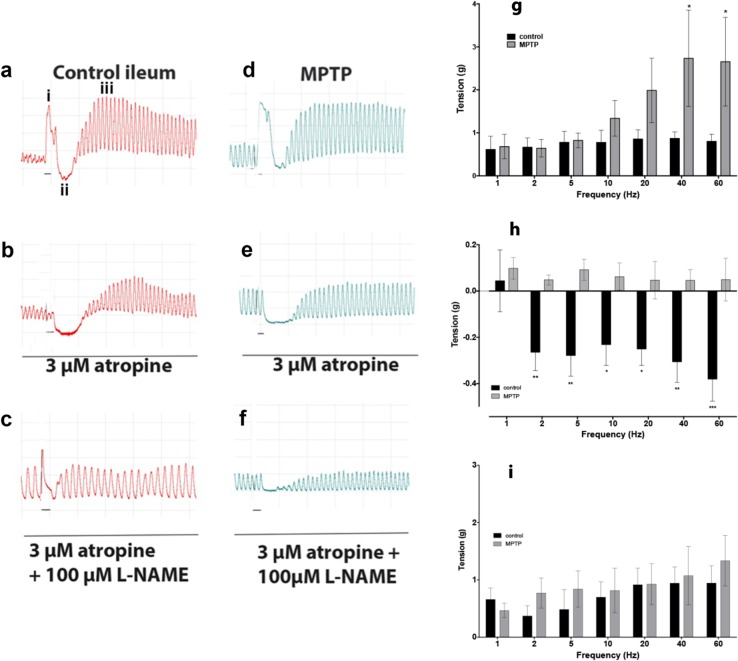
Characterisation of EFS-evoked contractile and relaxant responses of isolated common marmoset ileum. Representative traces showing the contractile response of isolated ileum from normal, drug naive (**a**–**c**) and MPTP treated common marmoset (**d**–**f**) in response electrical field stimulation and their pharmacological manipulation. Contraction elicited by 10 s trains of pulses at 10 Hz produced triphasic contractions **a**: initial contraction (phase-i) followed by a later appearing relaxation (phase-ii) and a slow developing contraction following the relaxation response (phase-iii). Phases i and ii were atropine **b** and L-NAME **c** sensitive, respectively. In tissues obtained from the MPTP treated animals component ii was markedly reduced (**d–f**). Panels (**g**–**i**) summarise the contractile and relaxation responses to EFS at differences stimulation frequencies: **g** phase-i; **h** phase-ii; **i** phase-iii. Small horizontal bars indicate the duration of EFS. Each bar is the mean ± s.e.m. of *n* = 6. *P < 0.05; **P < 0.001; ***P < 0.0005

**Fig. 3:**
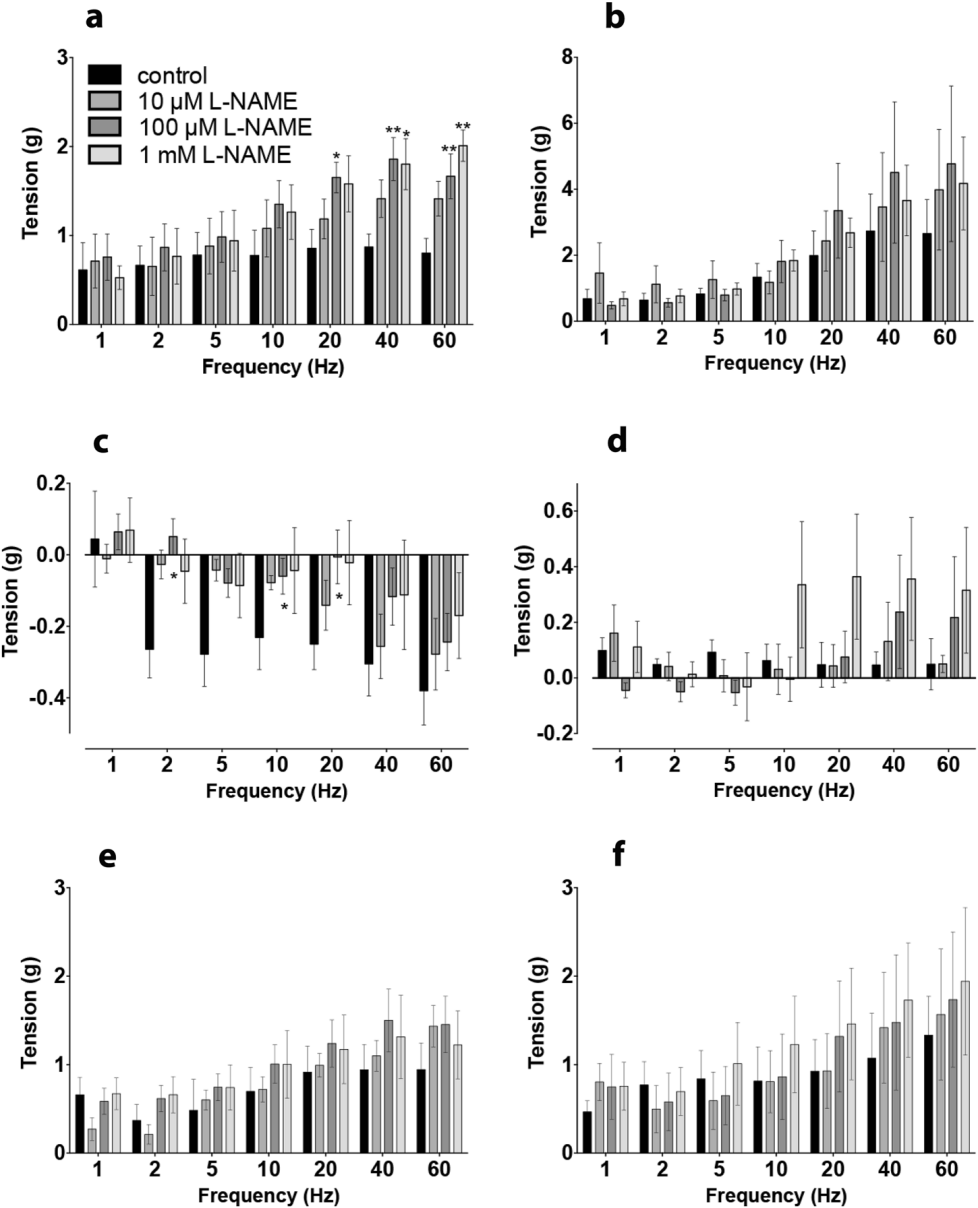
Contribution of nitrergic transmission to EFS-evoked contractile and relaxant responses of common marmoset ileum. The effect of L-NAME on the EFS-evoked contractile and relaxation response of the normal, drug naive (**a**, **c**, **e**) and MPTP-treated (**b**, **d**, **f**) common marmoset ileum. L-NAME concentration dependently increased the phase-i contractile response in normal **a** but had no effect on the ilea of the MPTP-treated animals **b**. The phase-i and the phase-ii relaxation was concentration dependently inhibited by L-NAME in the normal **c** but in tissues from the MPTP-treated animals, EFS-evoked relaxation was absent **d**. The phase-iii contractile response in tissues from normal **e** and MPTP-treated **f** were similar in magnitude but L-NAME did not have any significant effect on the EFS-evoked responses. Each bar is the mean ± s.e.m. of *n* = 6. *P < 0.05; **P < 0.001

Figure 2a–f shows representative response of normal and MPTP ileum to EFS at 10 s trains of pulses, at 10 Hz (50 V, 0.2 ms pulse width), the normal, control ileum produced a complex pattern of contraction/relaxation (Fig. 2a) which were composed of three distinct phases: phase-i: sharp contraction followed by phase-ii, long-lasting relaxation and phase-iii, and a tonic, slowly developing after contraction. Only, the initial phase-i was completely blocked by 3 µM atropine but phases-ii and -iii were unaffected (Fig. 2b). Combined administration of 3 µM atropine and 100 µM L-NAME completely blocked phases-i and -ii (Fig. 2c). In the ileum obtained from MPTP-treated marmosets, a similar contractile pattern was observed (Fig. 2d–f) but the relaxation component was almost absent (Fig. 2e). When the EFS-evoked responses at frequencies ranging from 1 to 60 Hz were examined, there was an overall frequency dependent increase in the phase-i responses in the tissues obtained from MPTP-treated animals, which at frequencies greater than 20 Hz were significantly greater with respect to the control tissues (Fig. 3g; *P* = 0.0049, *F*_(1–70)_ = 8.44; *n* = 6, 2-way ANOVA).

In response to EFS-evoked relaxation (phase-ii), the control ileum relaxation was evident only at frequencies higher than 2 Hz and these were maintained at 60 Hz. However, there was not a clear frequency dependent relaxation response (Fig. 3h; *P* = 0.116, *F*_(6,70)_ = 1.78; *n* = 6, 2-way ANOVA). In stark contrast to the normal tissues, the phase-ii responses of the ilea from the MPTP-treated animals were absent at all frequencies of stimulation examined beyond 2 Hz (*P* < 0.0001; *F*_(1,70)_ = 50.28, *n* = 6, 2-way ANOVA).

When the phase-iii responses were examined, there was a clear frequency-related enhancement of this late-appearing ‘after-contraction’ but there was no difference in the magnitude of the response between the control and the MPTP tissues (Fig. 2i).

Administration of NOS inhibitor L-NAME concentration dependently increased the force of contraction highly significantly in the normal ileum preparations (Fig. 3a; *P* = 0.0004; *F*_(3,140)_ = 6.40, *n* = 6, 2-way ANOVA). However, in the tissues from the MPTP-treated animals L-NAME had no significant effect overall (Fig. 3b; *P* = 0.506; *F*_(3,140)_ = 0.78; *n* = 6, 2-way ANOVA). When the relaxant component of the response (phase-ii) was investigated, it was shown that L-NAME concentration dependently inhibited the phase-ii responses only in the tissues obtained from the normal animals (Fig. 3; *P* = 0.0003; *F*_(3,140)_ = 6.81; *n* = 6, 2-way ANOVA) but in those obtained from the MPTP-treated animals very little EFS-evoked relaxation was seen. Instead, L-NAME at higher (>100 µM) concentration seemed to reverse the relaxation to modest contractions (Fig. 3d). However, the effect of L-NAME on phase-ii component did not quite reach the level of significance (*P* = 0.0541; *F*_(3,140)_ = 2.61, *n* = 6, 2-way ANOVA).

There was a frequency-related increase in the magnitude of the phase-iii ‘after contractions’. However, L-NAME at any concentration used had no effect on tissues obtained either from the normal or the MPTP-treated animals (Fig. 3e; control: *P* = 0.0765; *F*_(3,140)_ = 2.33; Fig. 3f; MPTP: *P* = 0.504; *F*_(3,140)_ = 0.79; *n* = 6; 2-way ANOVA).

### Immunohistochemical analysis of myenteric plexus

**Fig. 4:**
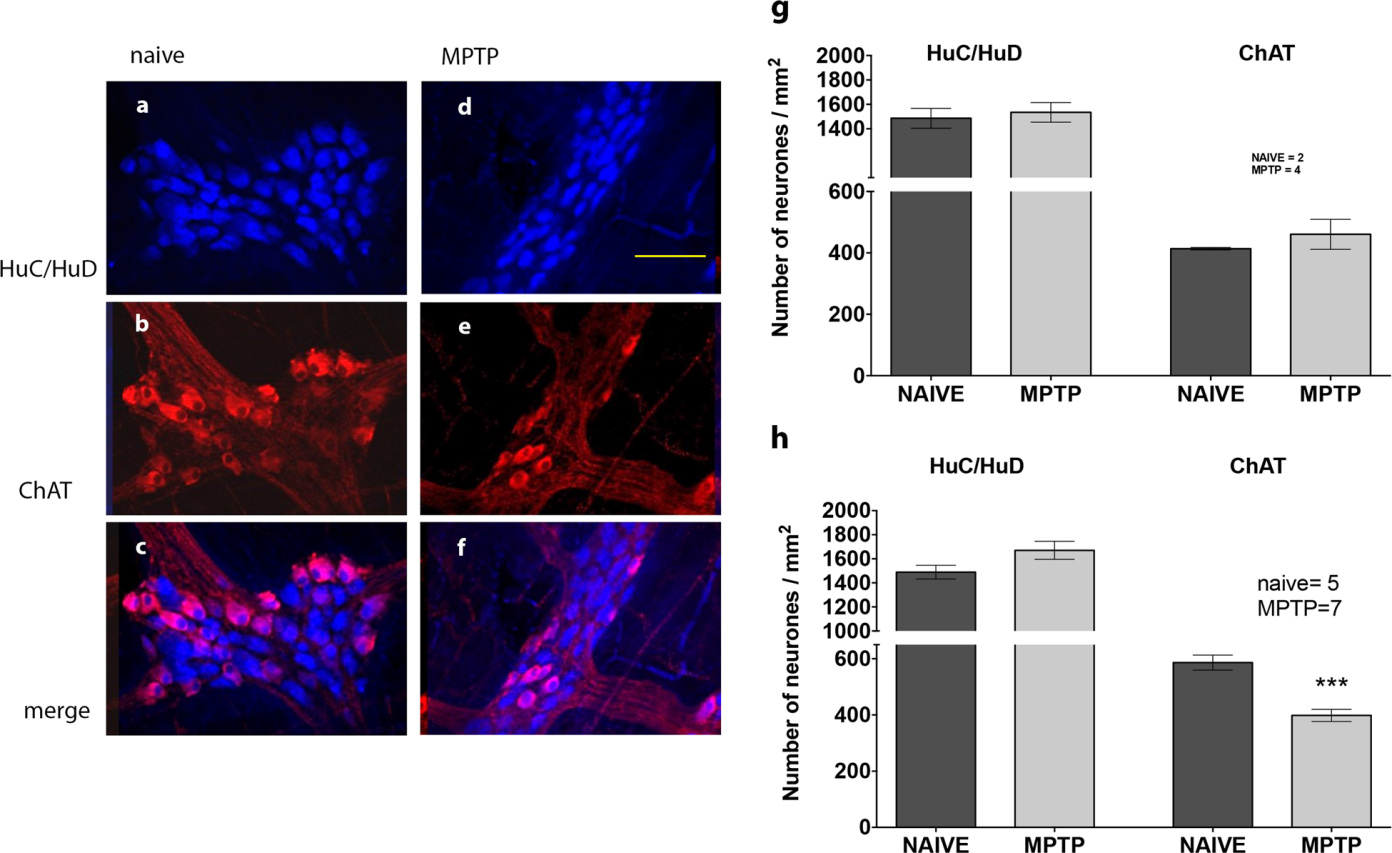
The effect of MPTP treatment on expression of cholinacetyltransferase-immunoreactivity (ChAT-ir) in myenteric plexus of ileum. Quantitative analysis of HuC/HuD and ChAT-ir in the whole-mount myenteric plexus preparations from normal, drug naive **a**, **b**, **c** and MPTP-treated **d**, **e**, **f** animals. The number of HC/HuD is unchanged in proximal **g** and distal **h** ileum. In the proximal ileum **h**, ChAT-ir is unchanged but it is significantly reduced in the distal ileum myenteric plexus **g**; ***P < 0.0005. Scale bar (**d**) represents 100 µm

**Fig. 5:**
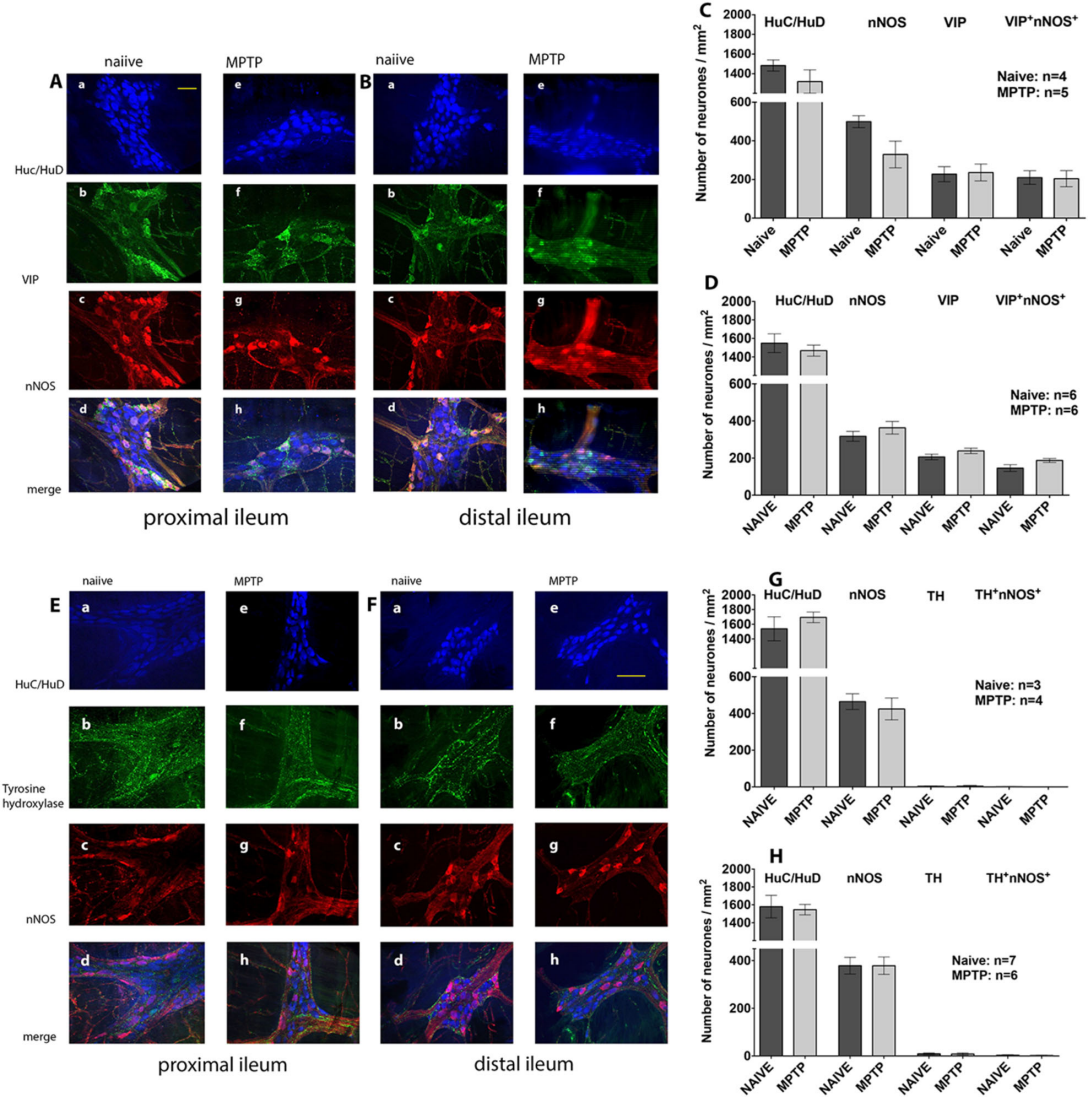
The effect of MPTP treatment on expression of VIP-ir, nNOS and TH-ir in myenteric plexus of ileum. Quantitative analysis of HuC/HuD and co-localisation of VIP- and nNOS-immunoreactivity (**A[a**–**h]**, **B[a**–**h]**) and Tyrosine hydroxylase (TH)- and nNOS immunoreactivity (**E[a**–**h]** and **F[a**–**h]**) in the whole mount myenteric plexus of proximal (**A[a**–**h]**, **E[a**–**h]**) and distal (**B[a**–**h]**, **F[a**–**h]**) and ileum preparation of normal, drug naive and MPTP-treated animals. The number of HC/HuD is unchanged in proximal (**c**, **g**) and distal (**d**, **h**) ileum. The individual number of nNOS-ir and VIP or the number of nNOS/VIP co-localisation and number of individual nNOS-ir and TH-ir or nNOS/TH-ir co-localisation was not changed in the proximal and distal ileum following MPTP-treatment. Scale bars represent 50 µm (Aa) and 100 µm (Ee)

**Fig. 6:**
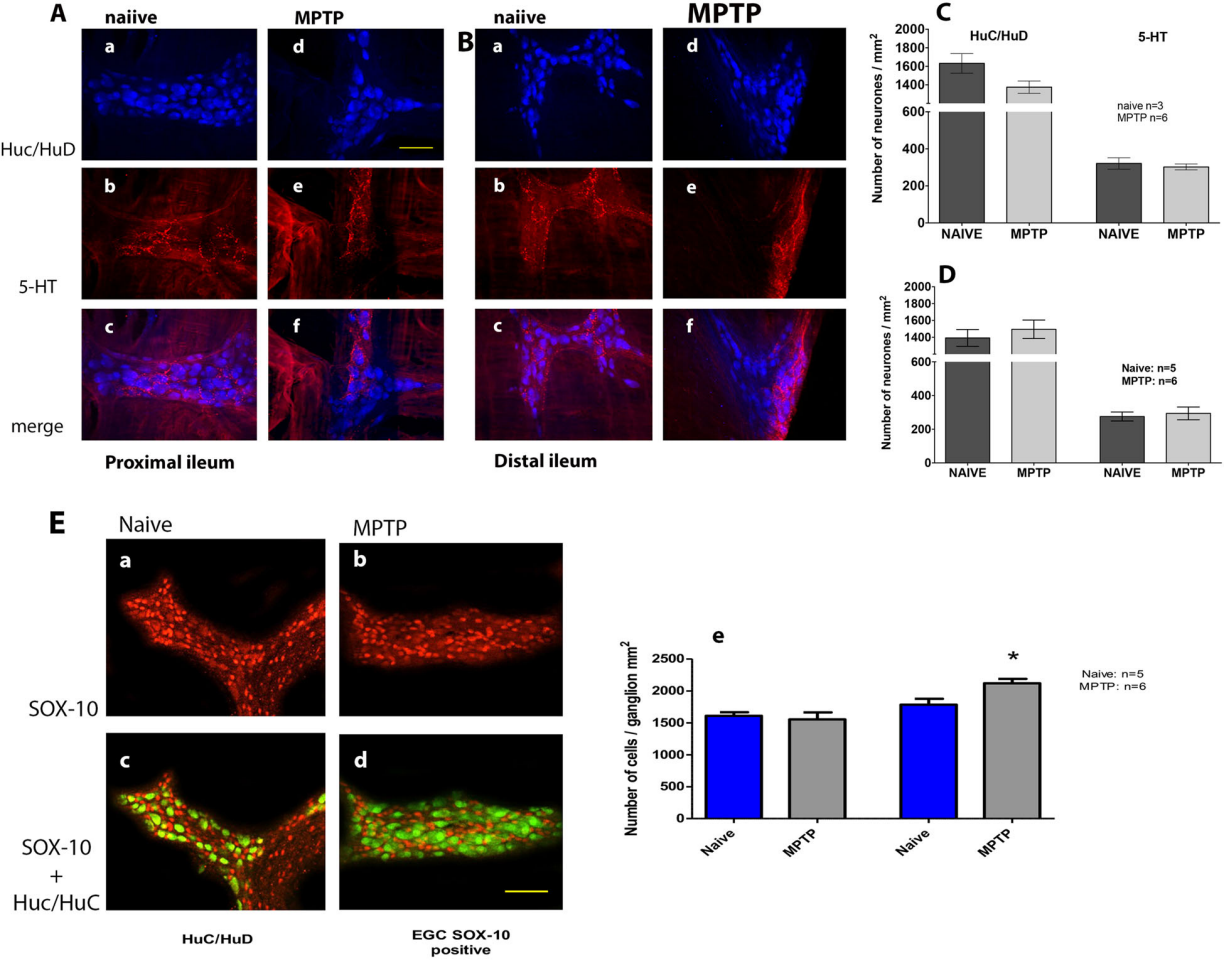
The effect of MPTP treatment on serotonergic fibres and on immune cells in the myenteric plexus of ileum. Quantitative analysis of HuC/HuD and 5-HT immunoreactivity (5-TH-ir) in the whole-mount myenteric plexus preparations from normal, drug naive (**A[a**–**c]**, **B[a**–**c]**) and MPTP-treated (**A[d**–**f]**, **B[d**–**f]**) animals. The number of HC/HuD is unchanged in proximal **c** and distal **d** ileum and MPTP treatment did not affect the expression of 5-HT-ir expression in either region of the ileum. In **e**, quantitative analysis of HuC/HuD and SOX-10 immunoreactive glial cells (SOX-10-ir) in the whole-mount myenteric plexus preparations from normal, drug naive and MPTP-treated distal ilea (**a**–**d**) is shown. There was a similar number of HuC/HuD and SOX-ir cells in the myenteric plexus **e** but following MPTP treatment there was a modest but significant increase in the number of SOX-ir; *P < 0.05. Each bar is the mean ± s.e.m. Scale bar represent 100 µm

Samples of proximal and distal ileum from normal and MPTP-treated animal showed that there was no significant difference in the total number of neurones as assessed by counting the total number HuC/HuD immunoreactivity per ganglia/mm^2^. This was a consistent pattern of observation from all samples studied (Figs 4–6). In the proximal ileum, no difference was observed between the number of ChAT-ir neurones colocalised with HuC/HuD-ir neurones in the normal and MPTP tissues (Fig. 4g). However, the number of normal controls were too few to allow meaningful statistical analysis. In the distal ileum however, there was a modest but highly significant reduction of the number of ChAT-ir neurones (Fig. 4c–f) that were colocalised with HuC/HuD in MPTP tissues (Fig. 4h; *P* = 0.0005; two-tailed Student’s unpaired *t*-test *n* = 5–7).

When we compared the number of nNOS-ir and VIP-ir neurones in the proximal (Fig. 5a) and the distal (Fig. 5b) ileum of normal and MPTP-treated animals, we found that majority, but not all, of the nNOS-ir and VIP-ir neurones were colocalised in both the normal (Fig. 5A[a–d], B[a–d]) and MPTP (Fig. 5A[e–h], B[e–h]) ilea. Since there were a few nNOS-ir and VIP-ir neurones that were not colocalised, cell counts were therefore expressed as individual counts for nNOS-ir and VIP-ir respectively or when nNOS-ir and VIP-ir were colocalised. The cell count data for the proximal and distal ilea are shown in Fig. 5c, d, respectively. There was no difference in the total number of nNOS-ir neurones in the proximal or the distal ileum of the MPTP-treated animal (Fig. 5c, *P* = 0.0795; unpaired Student’s *t*-test; *n* = 4–5; Fig. 5d, *P* = 0.312, *n* = 6). The number of colocalised nNOS/VIP-ir was counted, were smaller in both normal and MPTP of both proximal and distal ilea, but there were no significant differences between them (Fig. 5c, *P* = 0.921, *n* = 4–5; Fig. 5d, *P* = 0.0813, *n* = 6).

We also looked at the pattern of distribution of TH-ir in the myenteric plexus. TH-ir was localised to fine fibres surrounding the Huc/HuD-ir cells in the proximal (Fig. 5E[b–f]) and distal ileum (Fig. 5F[b–f]). There was a generalised reduction in the intensity of TH-ir staining following MPTP (Fig. 5E[f], F[f]), but it was not possible to accurately quantify the extent of reduction of TH-ir using the currently used protocols. Triple labelling for HuC/HuD-ir, TH-ir and nNOS-ir in both proximal and distal ileum showed that TH-ir fibres surrounded some of the HuC/HuD-ir cells, a few of which were also nNOS-ir. There was no difference in the number of HuC/HuD-ir neurones that were associated with TH-ir and the number of Huc/HuD-ir neurones that were colocalised with nNOS and were associated with TH-ir fibres and were not different in the proximal and the distal ilea of the MPTP-treated animals (Fig. 5g, h, respectively).

In a similar fashion to TH-ir, 5-HT-ir fibres also finely arborised around HuC/HuD-ir cells within the myenteric plexus ganglia in the proximal (Fig. 6A[b–f]) and distal ileum (Fig. 6B[b–f]). Many but not all 5-HTir fibres were associated with Huc/HuD cells in both regions of normal and MPTP ileum. However, unlike TH-ir, no evidence of any change in 5-HT-ir fibres was observed in different regions and following MPTP treatment.

Using a SOX-10 antibody, a reliable marker of the enteric glial cells,^14^ we found extensive immunoreactivity in the distal ileum but there was no colocalisation of SOX-10-ir with HuC/HuD-ir cells in any region investigated (Fig. 6E[a–d]). However, when the number of SOX-10-ir cells were counted, there was a modest but statistically significant increase in SOX-10-ir in the distal ileum myenteric plexus of the MPTP-treated animals (Fig. 6E[e]). Not enough samples were available to perform SOX-10-ir in the proximal ileum.

## Discussion

Gut dysmotility is a common problem in PD and it can be reproduced in several rodent and primate models of PD where the dopaminergic nigro-striatal pathway has been destroyed.^1,6–9^ However, very little is known about the mechanisms that initiate the abnormalities in the motility of the gastrointestinal tract. Because toxin-induced ablation of the nigrostriatal tract in animals results in similar gut dysfunction to that seen in PD, a role for the central dopaminergic system is suggested that may in turn induce an indirect effect on the dorsal motor nucleus of vagus and vagal outflow. Indeed, direct administration of 6-OHDA into the dopaminergic SN results in the loss of cholinergic and nitrergic neurones in the dorsal vagal complex.^8^ Alternatively, direct alterations in gut function and morphology may occur as pathological markers associated with PD, in the form of α-synuclein accumulations, are present in the gut.

Indeed, the results of the present study suggest that there are marked local contractile changes occurring in the gut following MPTP-treatment of common marmosets. The changes in contractility did not appear to be due to altered receptor–effector coupling as the responsiveness to the cholinergic agonist, carbachol and to histamine or to the relaxing effects of NA did not differ between the tissues obtained from the control and the MPTP treated animals. Using the EFS parameters chosen, a range of contractile responses could be seen. The primary phasic contraction which was blocked entirely by atropine (phase-i) was therefore deemed to be entirely cholinergic; a later appearing relaxation which was blocked almost entirely by the NOS-inhibitor L-NAME, so with a high level of confidence this component was attributed to the generation and the release of NO (phase-ii). The after-contraction response (phase-iii) is more difficult to explain. While the magnitude of the delayed contractile response appeared to be frequency dependent, but lack of modulation of these responses by either atropine or L-NAME suggested that the combination of excitatory and inhibitory transmitters was not involved. It is plausible that the after-contraction may be due to physiological adjustments of the longitudinal smooth muscle cells following EFS-induced release of excitatory and inhibitory neurotransmitters. However, interestingly the lack of significant differences between the tissues from the normal and the MPTP animals suggest that the observation of the differences in phase-1 and phase-ii responses were not due to the differences in muscular mass of ilea from these animals.

At frequencies of stimulation higher than 20 Hz, the ilea from the MPTP-treated animals contracted to a significantly greater extent compared to the controls and the relaxation in response to EFS was virtually absent at all stimulation frequencies. Both observations could easily be explained by the loss of intrinsic nitrergic transmission. It has previously been shown that in normal guinea-pig ileum, inhibition of nitric oxide synthase could have two important effects: Firstly, inhibition of NO synthesis which will not only prevent the NO-mediated relaxation^12^ but may also indirectly enhance EFS-evoked contractile response because the contribution to EFS to contraction will likely result from release of both excitatory (ACh) and inhibitory (NO) and inhibition of NO synthesis will allow only the expression of the effects of the excitatory neurotransmitter. Secondly, NO has been shown to inhibit ACh release,^13–16^ so inhibition of NOS will allow more EFS-evoked released of ACh. There are several possible explanations as to why the contractile responses at higher EFS frequencies might be greater in the MPTP tissues. Since EFS is likely to depolarise all nerve endings indiscriminately, then increasing the strength of stimulation at higher frequencies is likely to recruit deeper and finer nerve inhibitory fibres more readily. Activation of these fibres may release both NO or another inhibitory neurotransmitter VIP which in turn would control ACh release. Because these fibres may require more intense stimulation, then dysfunction of these fibres following MPTP treatment would reveal the enhanced contraction at higher EFS frequencies only in the MPTP ileum. Thus, we propose that the dysfunction of the nitrergic transmission is the likely cause of enhanced contractile response of the longitudinal smooth muscle of the ileum following MPTP. That, phase-ii responses were already inhibited at all stimulation frequencies, suggest that NO may indeed have two distinct functions as stated above. Another evidence for nitrergic dysfunction following MPTP comes from the effects of L-NAME. The NOS inhibitor L-NAME concentration dependently potentiated the phase-i contractions of the control tissue, again at frequencies above 20 Hz to a similar magnitude as the MPTP tissues, but in the absence of L-NAME. However, L-NAME had no effect on the phase-i response of the tissues from MPTP treated animals. Moreover, the EFS-evoked phase-ii relaxation of the control tissues was also concentration dependently inhibited by L-NAME had no effect on the phase-ii response of the MPTP ileum. Indeed, there was some evidence of rebound contraction as the concentration of L-NAME was increased. Nitrergic dysfunction in PD is not unprecedented. One interesting parallel which circumstantially supports the notion of nitrergic dysfunction in PD is the occurrence of erectile dysfunction in PD^17^ which in a similar to gut dysfunction can be ameliorated by apomorphine. However, since the intrinsic neurotransmitter involved in penile erection is NO, then and inhibition of the phosphodiesterase-V can enhance the effects of the residual levels of NO on cyclic GMP could reverse erectile dysfunction. Consequently, agents such as sildenafil that enhance the level of cyclic GMP might have some utility in the treatment of bowel dysfunction in PD. Indeed, recently the efficacy of sildenafil in normalising gut transit in a mouse model of constipation has been demonstrated.^18^

Similar contractile and relaxant responses to the present study was also reported earlier in the colon of MPTP treated mice^6^ but curiously, the authors of that study compared the mechanical response of the colon with NADPH-diaphorase staining in the ileum and they could not find any loss of nitrergic innervation in the myenteric plexus. However, this was in contrast to a study in 6-OHDA lesioned rats^9^ where the contractile response and ChAT-ir of isolated colon was reduced compared to controls. Interestingly, a study comparing the neurotransmitter phenotypes along the GI tract of macaques and human and another in PD showed a significant overlap between NOS and VIP along the myenteric plexus of these two species, but there was an increasing gradient in the expression of NOS-ir neurones from the stomach down to the rectum.^19,20^ However, this gradient was reversed for ChAT-ir.^19^ This finding strongly suggests that the expression of different neurotransmitters is not uniform and any findings with regards to the extent of cholinergic and nitrergic innervation in the ileum does not apply to the colon. Moreover, those studies suggest that locations along the GI tract where samples are taken greatly influences the observed expression of various neurotransmitter markers. Thus, it might be the reason why different results have been seen in different studies. For this reason, we only looked at the expression of several neurotransmitters along the proximal and distal ileum and consequently what we found cannot be extrapolated to the colon where most of the previous investigations were conducted.

In confirmation of earlier study in PD^20^ we found no evidence of loss of myenteric ganglion neurones as revealed by Huc/HuD-ir and in confirmation of the findings of Anderson et al.,^6^ in the MPTP-treated mice and the intranigral 6-OHDA administration^8^ we found that the expression of ChAT-ir was significantly reduced only in the distal ileum of the MPTP-treated animals, from where the tissues samples used for the functional study were obtained. Our VIP-ir and NOS-ir studies showed that nearly all VIP-immunoreactivity was colocalised with NOS. However, most interestingly we found no change following MPTP treatment in either the NOS-ir or the VIP-ir in any part of the ileum suggesting that the inhibitory transmitters were completely intact. This finding also mirrored the observations made in MPTP treated mice,^6^ MPTP-treated primates^5^ and in human PD.^20^ This finding is quite intriguing because our functional data clearly suggested that there is an impairment of the nitrergic system following MPTP treatment found no evidence of alterations in NOS-ir. Anderson et al.^6^ suggested that the enhanced contraction and the impaired relaxation of the smooth muscle of colon from MPTP-treated mice in response to EFS may result from the loss dopaminergic innervation of the myenteric plexus. Since TH-ir was confined, at least in this case to fine nerve fibres, it was very difficult to quantify any changes following MPTP. Consequently, we counted all the Huc/HuD-ir neurones that were closely associated with TH-ir. Based solely on qualitative observation of the TH-ir alone, there was an indication of reduced intensity of TH-ir fibres in the myenteric plexus, but when the association of TH-ir fibres with Huc/HuD-immunoreactive cells were assessed we found no significant difference between tissues obtained from the normal and MPTP-treated animals. Moreover, compared to other neurotransmitters, very little dopamine is present in the myenteric plexus. Although there is evidence that in neurotoxin-induced parkinsonism in rodent and in PD there is a distinct loss dopaminergic innervation^5,6,21–23^ but this finding has not been consistent.^20^ Consequently, we feel that the role of dopamine in the observed contractile response of the MPTP ileum is unlikely. There was far more 5-HT-ir fibres associated with HuC/HuD fibres compared to TH-ir but again there was no difference in 5-HT-ir fibres between the normal and the MPTP ilea.

Chaumette et al.^5^ showed that there was no change following MPTP treatment in the enteric glial inflammatory marker, SOX-10^24^ in macaques. Their assessment was based on counting the number of SOX-10-ir cells within the myenteric ganglia from four animals. Here, in 5 control and 6 MPTP animals we found a modest but statistically significant increase in the number of SOX-10-ir cells, which suggested that MPTP treatment led to inflammation of the ileum. While it is known that in the brain or in the ileum, EFS evokes NO release^14,15,25^ and inflammatory cells in the brain (microglia and astrocytes) are also an important source of nitric oxide,^26,27^ but it is not known whether EFS is likely to cause the release of non-neuronal NO from either the brain or the enteric glial cells. Moreover, NOS-inhibitors currently do not have sufficient selectivity to distinguish the contribution of different iso-forms of NOS towards NO release. Consequently, on the balance of probabilities, EFS-evoked release of NO from eNOS or iNOS is unlikely.

In conclusion, the pharmacological results of this study that the contractile response of the longitudinal smooth muscle of the ileum is significantly larger and the relaxation is absent in response to EFS strongly suggests that central dopaminergic denervation following MPTP treatment leads to dysregulation of nitrergic transmission, which will indirectly enhance the contraction of the longitudinal muscle but will prevent it from relaxing. This has the effect of disrupting peristaltic movement which is likely to slow down gut motility. However, there is a caveat to the interpretation of the data in that we have not monitored the peristaltic activity and using this experimental set up we can only draw the conclusions related only to the activity of the longitudinal muscle. Thus, the effect of MPTP-treatment on the activity of the circular muscle in this animal model remains untested and unknown. Nevertheless, the importance of ileal dysmotility in the onset of constipation cannot be ignored as several studies have indicated that ileum is involved^11,12^ although pathologies that lead to slow transit constipation are similar in both ileum and colon.^28^ The clinical correlates of these changes, if any, are unknown, although the very uncomfortable sensation of abdominal bloating experienced by some individuals with PD, especially as an ‘off’ phenomenon, could be the consequence of small-intestinal dysmotility.^29^ Further work would be needed to tease out the mechanism(s) involved in this process.

## Methods

### Animals

All experimental work was carried out in accordance with the Animals (Scientific Procedures) Act 1986 approved by the Kings College London Ethical Review Committee. In particular, the primate experiments reported were subject to and were carried out under the Animals (Scientific Procedures) Act 1986 under a Home Office Project Licence (PPL 70/4986) approved by the King’s Ethical Committee which complied fully with the guidelines and recommendations set out in the Weatherall Report 2006—the use of non-human primates in research (https://royalsociety.org/policy/publications/2006/weatherall-report/). The animals’ environment was enriched by installation of viewing turret on top of the cages to mimic height as would be the case in a normal habitat (height: 36 cm, width: 35 cm, depth: 50 cm) and wooden ladders/perches, Hammocks, swings, nesting boxes, multiple feeding platforms and saw dusted floors for forage feeding.

Two groups of normal controls (*n* = 7; 3 males and 4 females, 333–407 g; 373 ± 16 g) and 1-methyl-4-phenyl-1,2,3,6-tetrahydropyridine (MPTP; Sigma, Poole, UK)-treated (*n* = 7; 3 males and 4 females, 313–389 g; 359 ± 17 g) (MPTP, 5 × 2 mg/kg s.c.) adult common marmosets (*Callithrix jacchus*, Harlan UK Ltd. Loughborough, LE12 9TE, UK, Manchester University and King’s College London) were used in this study. The MPTP-treated animals were prepared according to the previously published protocols^30–32^ and were used in other studies where the symptomatic effects of various dopamine agonists were examined. MPTP-lesioned animals were culled between 1 and 3 years after the end of MPTP treatment, so there was no likelihood of the presence of residual MPTP. The normal marmosets were completely drug and toxin naive. Similarly treated animals exhibited greater than 70% loss of dopaminergic neurons in the SN.^33^

All marmosets were kept in home cages with dimensions of height: 166, width: 140 and depth: 90 cm at an ambient temperature of 25 ± 1 °C. Animals were housed in home cages in pairs, as approved by the Home Office inspectorate at King’s College London facilities, in a 12 h light/dark cycle at an ambient temperature of 25 ± 1 °C and were fed once daily with a diet of bananas, oranges and apples and had free access to food pellets (Mini Marex-E; Special diet Services) and drinking water. The detail of animals used and their motor disability scores as well as their survival time after last MPTP treatment have been summarised in Table 1.

### Organ bath studies

Common marmosets were killed using overdose of pentobarbital sodium (60 mg/kg; Euthatal, Merial Animal Health Ltd.) between 7:30 and 8:30 am. Upon cessation of foot and corneal reflexes, the thoracic and abdominal cavities were opened. The animals were transcardially perfused with ice-cold oxygenated (95% O_2_ plus 5% CO_2_) Krebs–Henseleit solution (composition in mM: NaCl 118, KCI 4.7, CaCl_2_ 2.5, MgSO_4_ 1.2, NaHCO_3_ 25, KH_2_PO_4_ 1.2, glucose 11) and a length of the proximal to the distal ileum before the start of the descending colon was excised and placed in this solution. While still in aerated Krebs–Henseleit solution, four to five 2 cm lengths of the ileum were cut transversely through the lumen. The proximal and the distal segments saved for immunohistochemical analysis and the middle portions were transported to the laboratory where they were suspended in a 15 ml organ baths and placed under a resting tension of 1.0 g force at 37 °C. Following a 30–60 min equilibration period, and initiation of spontaneous contractions, the contractile activity of the ileum was measured using an isometric transducer connected to LabChart data acquisition system (AD Instruments Ltd., Oxford, UK).

Tissues viability was tested initially by the administration of carbachol (CCH), 10 µM to access the contractile responses. Only tissues that contracted >2 g tension were included in the study. To assess whether prior MPTP treatment affected the receptor/effector coupling or whether there were changes at the level of smooth muscle, the isolated ileum preparations were contracted by cumulative addition of various concentrations of CCh (0.01–30 µM; Sigma-Aldrich) or histamine (0.1–100 µM; Sigma-Aldrich). Tissues were then thoroughly washed with several changes of Kreb’s solution and subsequently the relaxant effect of noradrenaline (NA) was then compared on the amplitude, tone and the frequency the spontaneous contractile response of the ileum from naive and MPTP-treated animals. Since there is evidence for the presence of enteric dopaminergic neurones^34^ that modulate intestinal motility, which might be affected following MPTP-treatment, the effect of both dopaminergic D1 agonist, A77636 or D2 agonist ropinirole were tested on the tone, amplitude and frequency of spontaneous rhythmic contractions of the ileum preparation obtained from normal, drug naive or MPTP-treated animals. The amplitude was estimated by adding together the tension of individual contractions occurring during a period of 10 min and dividing this sum by the number of contractions during this period. The rate of spontaneous activity (rate/min) was derived from dividing the total number of contractions during the 10 min observation period.

### Electrical field stimulation induced contractions and relaxations

To assess any neurogenic alterations in the contractile and relaxant responses following MPTP treatment, ileum preparations were contracted indirectly by EFS delivered through a pair of platinum electrodes placed on either side of the muscle strips in the organ bath. The ileal segments from drug naive normal and MPTP-treated common marmosets were stimulated with 10 s trains of pulses at 1.0, 2.0, 5.0, 10, 20, 40 and 60 Hz and pulse duration of 0.2 ms at supramaximal voltage (50 V) once every 5 min. Contractile responses to EFS were evoked in the absence or in the presence of 3 µM atropine (atropine sulphate, Sigma-Aldrich) to assess the contribution of the cholinergic component to the EFS-evoked contractile responses. Atropine was administered 30 min before EFS-evoked responses started. To investigate the contribution of the NO to the overall contractile or relaxation responses following EFS, non-selective NOS inhibitor, L-nitroarginine methyl ester (L-NAME) at concentrations ranging 10 µM to 1 mM was added to the organ bath 15 min before EFS was effected.

### Nigrostriatal TH-immunoreactivity

In order to ascertain the extent of MPTP-induced nigrostriatal lesions, 30 µm free-floating sections from the SN and the striatum were processed for TH immunohistochemistry according to previously published protocol^35^ with some modifications regarding analysis of striatal TH+ve optical density in the caudate nucleus and the putamen. Briefly, following incubation with rabbit anti-TH antibodies (see Table 2) overnight at room temperature. Following colour development, The TH-immunoreactive (ir) neurones in the SN sections were manually counted. The number of TH-ir neurones at the level of the third nerve was derived by averaging 3–4 adjacent sections (depending on availability of the relevant sections) where the 3rd nerve rootlets were clearly identifiable. The striatal TH-ir at different regions following MPTP treatment was assessed by densitometry using Image-J software. Variations in staining intensity within the caudate and the putamen were assessed. To normalise for variation in staining intensity, a reading was obtained from the white matter in the corpus callosum and this was subtracted from readings obtained within the caudate nucleus and the putamen.

**Table 2:** The detail of primary antibodies used in this study and their host sources

Primary antibodies	Host	Dilution	Source
Neuronal protein Huc/HuD—monoclonal	Mouse	1:50	16A11; Molecular Probes
Vasoactive intestinal Polypeptide (VIP)—polyclonal	Rabbit	1:100	Sc-20727; Santa Cruz Biotechnology
Neuronal nitric oxide synthase (nNOS)—polyclonal	Rabbit	1:500	AB76442; Millipore
Tyrosine hydroxylase (TH)—polyclonal	Chicken	1:400	AB76442; Abcam
Tyrosine hydroxylase (TH)—polyclonal	Rabbit	1:1000	AB112; Abcam
Choline acetyl transferase (ChAT)—polyclonal	Goat	1:20	AB144P; Millipore
Serotonin (5-HT)—polyclonal	Rabbit	1:1000	PC228L; Merck Chemicals
SOX-10—polyclonal	Goat	1:100	Sc-17342; Santa Cruz Biotechnology

### Intestinal whole mount preparation

Proximal and distal segments of ileum were immersed for 15 min in 0.1 M phosphate buffered saline (PBS) pH 7.2 containing 1 µM nicardipine as a muscle relaxant (Sigma-Aldrich, UK). Sections were then opened along the mesenteric line and washed in PBS to remove the content of the lumen. Subsequently, the proximal and distal ileum preparations were stretched on a wax support with the mucosa layer facing downwards and fixed for 6/7 h in Zamboni’s fixative (2% paraformaldehyde containing 0.2% picric acid in 0.1 M PBS) at 4 °C. Following fixation, the tissues were removed from the wax support and washed in dimethylsulfoxide (DMSO) followed by three 10 min washes in PBS and then stored at 4 °C in PBS containing 0.1% sodium azide (Sigma-Aldrich). Stretch specimens were processed as longitudinal muscle-myenteric plexus whole mount preparations (LMMPs) by peeling away the different layers (i.e. mucosa, submucosa and circular muscle) as previously described.^2^

To analyse the enteric neurons in the myenteric plexus of the LMMPs, double staining (Hu/Chat, Hu/5-HT, Hu/Substance P and Hu/GFAP) or triple labelling (Hu/VIP/nNOS, Hu/TH/nNOS) immunofluorescence techniques were performed. Depending on the antibody characteristics either the immunofluorescence histochemistry, or labelled streptavidin–biotin (LSAB) methods were used.

### Double staining procedures

Prior to immunostaining, whole mount samples were permeabilised for 1 h at room temperature in blocking buffer containing 1% Triton X-100 (10% normal goat or donkey serum in 0.1 M PBS) to reduce non-specific binding. Whole mounts were then incubated overnight in mixtures of primary antibodies diluted in the blocking buffer, at 4 °C. Anti-Huc/HuD antibody was used to identify all myenteric neuronal cell bodies.^36^ Details of all primary antibodies used are summarised in Table 1. Whole mounts were then washed with PBS, and incubated with secondary antibodies diluted in the blocking buffer for 3 h at room temperature. The tissue preparations were then washed 3 × 10 min in PBS and then mounted onto slides using Mowiol mounting media (Mowiol 40-88 Sigma 324590, Tris Buffer 0.2 M pH 8.5, distilled water, Glycerol, DABCO-Sigma D-2522, Sodium Azide 10% stock).

### The labelled streptavidin–biotin (LSAB)

After staining with primary antibody, tissues were washed in PBS and then incubated with biotinylated secondary antibody, diluted in the blocking buffer, for 1 h at room temperature. Whole mount preparations were then incubated overnight with a second primary antibody following the same procedure for time and temperature. After 24 h, specimens were washed in PBS and incubated in fluorescent dye streptavidin and suitable secondary antibody for 3 h, then washing and mounting as described above.

The details of all the primary antibodies used and their sources are summarised in Table 2.

### Cell counting

Image capture was performed using a Zeiss ApoTome microscope and a Zeiss AxioCam camera. Images were captured by Zeiss AxioVision 4.0 image analysis software. Whole-mount preparations were used to perform a quantitative analysis of the different neuronal populations investigated (ChAT-ir, nNOS-ir, VIP-ir, 5-HT-ir, TH-ir and SOX-10-ir) with respect to the HuC/HuD-ir neuronal population. All observations were made at ×20 objective. Firstly, the number of neuronal cells counted within each ganglion in the myenteric plexus and this usually had an area of between 0.02 and 0.04 mm^2^ and approximately a total of about 250–300 HuC/HuD-ir neurones were counted from randomly selected ganglia. Normally 5 slides were counted per animal sample. Average counts for each animal was then normalised to total counts/mm^2^.

### Data analysis and statistics

For the amplitude and the frequency of spontaneous contractions, and the EFS-evoked contractions or relaxations at different stimulation frequencies the values for the replicates running in parallel (*n* = 2–3) were averaged for each animal and presented as *n* = 1. The overall mean ± s.e.m. was determined from the individual average values of all animals in their respective groups. Where multiple groups of data in concentration response curves or where the contractile or relaxant responses to different stimulation frequencies were compared, a two-tailed, two-way ANOVA followed by Fisher’s LSD multiple comparison post hoc test was used. For comparison of cell counts and immunohistochemical analyses expressed as total counts/mm^2^ were means expressed as mean ± s.e.m. and the differences between naive controls and MPTP samples were compared using an un-paired, two-tailed Student’s *t*-test. Where there was a correlation of dopaminergic cell loss with motor behaviour or gut function, data were analysed using a one-tailed Pearson’s *r* correlation. All statistical analyses were carried out using Prism 6.0 software (GraphPad, San Diego, CA USA). Differences were considered statistically significant at *P* < 0.05.

## Data Availability

The data sets generated during and/or analysed during the current study are available from the corresponding author on reasonable request and are available in the Figshare repository: https://figshare.com/s/d7d6b8487a1546a6a0c9.

## References

[1] Schapira AHV, Chaudhuri KR, Jenner P (2017). Non-motor features of Parkinson disease. Nat. Rev. Neurosci..

[2] Blandini F (2009). Functional and neurochemical changes of the gastrointestinal tract in a rodent model of Parkinson’s disease. Neurosci. Lett..

[3] Colucci M (2012). Intestinal dysmotility and enteric neurochemical changes in a Parkinson’s disease rat model. Auton. Neurosci..

[4] Zhang X (2015). Alteration of enteric monoamines with monoamine receptors and colonic dysmotility in 6-hydroxydopamine-induced Parkinson’s disease rats. Transl. Res..

[5] Chaumette T (2009). Neurochemical plasticity in the enteric nervous system of a primate animal model of experimental Parkinsonism. Neurogastroenterol. Motil..

[6] Anderson G (2007). Loss of enteric dopaminergic neurons and associated changes in colon motility in an MPTP mouse model of Parkinson’s disease. Exp. Neurol..

[7] Drolet RE, Cannon JR, Montero L, Greenamyre JT (2009). Chronic rotenone exposure reproduces Parkinson’s disease gastrointestinal neuropathology. Neurobiol. Dis..

[8] Toti L, Travagli RA (2014). Gastric dysregulation induced by microinjection of 6-OHDA in the substantia nigra pars compacta of rats is determined by alterations in the brain-gut axis. Am. J. Physiol. Gastrointest. Liver Physiol..

[9] Fornai M (2016). Enteric dysfunctions in experimental Parkinson’s disease: alterations of excitatory cholinergic neurotransmission regulating colonic motility in rats. J. Pharmacol. Exp. Ther..

[10] Ellett LJ (2016). Restoration of intestinal function in an MPTP model of Parkinson’s disease. Sci. Rep..

[11] Panagamuwa B, Kumar D, Ortiz J, Keighley MR (1994). Motor abnormalities in the terminal ileum of patients with chronic idiopathic constipation. Br. J. Surg..

[12] Bassotti G (2006). Enteric neuropathology of the terminal ileum in patients with intractable slow-transit constipation. Hum. Pathol..

[13] Hryhorenko LM, Woskowska Z, Fox-Threlkeld JA (1994). Nitric oxide (NO) inhibits release of acetylcholine from nerves of isolated circular muscle of the canine ileum: relationship to motility and release of nitric oxide. J. Pharmacol. Exp. Ther..

[14] Hebeiss K, Kilbinger H (1996). Differential effects of nitric oxide donors on basal and electrically evoked release of acetylcholine from guinea-pig myenteric neurones. Br. J. Pharmacol..

[15] Hebeiss K, Kilbinger H (1998). Nitric oxide-sensitive guanylyl cyclase inhibits acetylcholine release and excitatory motor transmission in the guinea-pig ileum. Neuroscience.

[16] Mang CF, Truempler S, Erbelding D, Kilbinger H (2002). Modulation by NO of acetylcholine release in the ileum of wild-type and NOS gene knockout mice. Am. J. Physiol. Gastrointest. Liver Physiol..

[17] Chaudhuri KR, Schapira AH (2009). Non-motor symptoms of Parkinson’s disease: dopaminergic pathophysiology and treatment. Lancet Neurol..

[18] Sharman SK (2017). Sildenafil normalizes bowel transit in preclinical models of constipation. PLoS ONE.

[19] Noorian AR, Taylor GM, Annerino DM, Greene JG (2011). Neurochemical phenotypes of myenteric neurons in the rhesus monkey. J. Comp. Neurol..

[20] Annerino DM (2012). Parkinson’s disease is not associated with gastrointestinal myenteric ganglion neuron loss. Acta Neuropathol..

[21] Singaram C (1995). Dopaminergic defect of enteric nervous system in Parkinson’s disease patients with chronic constipation. Lancet.

[22] Tian YM (2008). Alteration of dopaminergic markers in gastrointestinal tract of different rodent models of Parkinson’s disease. Neuroscience.

[23] Natale G (2010). MPTP-induced parkinsonism extends to a subclass of TH-positive neurons in the gut. Brain Res..

[24] Hoff S (2008). Quantitative assessment of glial cells in the human and guinea-pig enteric nervous system with an anti-SOX 8/9/10 antibody. J. Comp. Neurol..

[25] Iravani MM, Millar J, Kruk ZL (1998). Differential release of dopamine by nitric oxide in subregions of rat caudate putamen slices. J. Neurochem..

[26] Iravani MM, Kashefi K, Mander P, Rose S, Jenner P (2002). Involvement of inducible nitric oxide synthase in inflammation-induced dopaminergic neurodegeneration. Neuroscience.

[27] Iravani MM, Liu L, Rose S, Jenner P (2004). Role of inducible nitric oxide synthase in N-methyl-d-aspartic acid-induced strio-nigral degeneration. Brain Res..

[28] Knowles CH, Farrugia G (2011). Gastrointestinal neuromuscular pathology in chronic constipation. Best Pract. Res. Clin. Gastroenterol..

[29] Pfeiffer RF (2003). Gastrointestinal dysfunction in Parkinson’s disease. Lancet Neurol..

[30] Smith L, De Salvia M, Jenner P, Marsden CD (1996). An appraisal of the antiparkinsonian activity of piribedil in 1-methyl-4-phenyl-1,2,3,6-tetrahydropyridine-treated common marmosets. Mov. Dis..

[31] Iravani MM, Tayarani-Binazir K, Chu WB, Jackson MJ, Jenner P (2006). In 1-methyl-4-phenyl-1,2,3,6-tetrahydropyridine-treated primates, the selective 5-hydroxytryptamine 1a agonist (R)-(+)-8-OHDPAT inhibits levodopa-induced dyskinesia but only with increased motor disability. J. Pharmacol. Exp. Ther..

[32] Jackson MJ, Smith LA, Al-Barghouthy G, Rose S, Jenner P (2007). Decreased expression of l-dopa-induced dyskinesia by switching to ropinirole in MPTP-treated common marmosets. Exp. Neurol..

[33] Pritchard S (2017). Altered detrusor contractility in MPTP-treated common marmosets with bladder hyperreflexia. PLoS ONE.

[34] Li ZS, Schmauss C, Cuenca A, Ratcliffe E, Gershon MD (2006). Physiological modulation of intestinal motility by enteric dopaminergic neurons and the D2 receptor: analysis of dopamine receptor expression, location, development, and function in wild-type and knock-out mice. J. Neurosci..

[35] Iravani MM, Haddon CO, Cooper JM, Jenner P, Schapira AH (2006). Pramipexole protects against MPTP toxicity in non-human primates. J. Neurochem..

[36] Szabo S (1991). Gastroduodenal mucosal injury—acute and chronic: pathways, mediators, and mechanisms. J. Clin. Gastroenterol..

